# Supplementary material for: The decline and fall of the mammalian stem
Source: PeerJ. 2024 Feb 27;12:e17004. doi: 10.7717/peerj.17004 (PMC10906263; doi:10.7717/peerj.17004)
Supplement: Data S2 — Deletions and modifications to the original PBDB occurences, and their justifications [file peerj-12-17004-s002.docx]

Removed occurrences 1559743, 898230, 792144, 990504, 780906, 1401731, 1400934, 381173, 575516, 1426192, 1400940 and 1400939 (appear to be footprint records although recorded as body fossils; not included in this analysis)

Removed occurrence number 841557 (Cretaceous dicynodont), now believed to be late Cenozoic mammal (Knutsen & Oerlamans 2020).

Removed occurrence number 1455756 (Langobarian therian); publication cited makes no mention of it; no record found in published literature of a therian at Espejeras, locality and formation are primarily marine invertebrates.

Occurrence 283938 and 283945 reassigned to *Kuehneotherium* (Stem mammals); (Marzola et al 2018)

Occurrence 921143 (*Tikitherium*) reassigned to stem mammals (Luo & Martin 2007; Panciroli et al 2021)

Occurrence 636937 deleted; Original record of an unnamed tooth. Later assigned to *Stirtodon*, but in the PBDB a separate occurrence was created (Clemens et al 2003; Rich et al 2020.

Occurences 380704 and 380705 deleted; Original records of unnamed teeth that were later assigned to *Theroteinus nikolai*, but in the PBDB a separate occurrence was created (Sigogneau-Russell et al 1986; Debuysschere 2016)

Occurrences 846763, 1061295, 1061296 (*Woutersia*) reassigned to stem mammals (Luo & Martin 2007)

References

Clemens, W. A., Wilson, G. P., & Molnar, R. E. (2003). An enigmatic (synapsid?) tooth from the Early Cretaceous of New South Wales, Australia. Journal of Vertebrate Paleontology, 23(1), 232-237.

Knutsen, E. M., & Oerlemans, E. (2020). The last dicynodont? Re-assessing the taxonomic and temporal relationships of a contentious Australian fossil. Gondwana Research, 77, 184-203.

Luo, Z. X., & Martin, T. (2007). Analysis of molar structure and phylogeny of docodont genera. Bulletin of Carnegie Museum of Natural History, 2007(39), 27-47.

Marzola, M., Mateus, O., Milan, J., & Clemmensen, L. B. (2018). A review of Palaeozoic and Mesozoic tetrapods from Greenland. Bulletin of the Geological Society of Denmark, 66, 21-46.

Panciroli, E., Benson, R. B., Fernandez, V., Butler, R. J., Fraser, N. C., Luo, Z. X., & Walsh, S. (2021). New species of mammaliaform and the cranium of *Borealestes* (Mammaliformes: Docodonta) from the Middle Jurassic of the British Isles. Zoological Journal of the Linnean Society, 192(4), 1323-1362.

Rich, T. H., Flannery, T. F., & Vickers-Rich, P. (2020). Evidence for a remarkably large toothed-monotreme from the Early Cretaceous of Lightning Ridge, NSW, Australia. Biological Consequences of Plate Tectonics: New Perspectives on Post-Gondwana Break-up–A Tribute to Ashok Sahni, 77-81.
